# Unusual Deformation and Fracture in Gallium Telluride Multilayers

**DOI:** 10.1021/acs.jpclett.2c00411

**Published:** 2022-04-25

**Authors:** Yan Zhou, Shi Zhou, Penghua Ying, Qinghua Zhao, Yong Xie, Mingming Gong, Pisu Jiang, Hui Cai, Bin Chen, Sefaattin Tongay, Jin Zhang, Wanqi Jie, Tao Wang, Pingheng Tan, Dong Liu, Martin Kuball

**Affiliations:** † State Key Laboratory of Superlattices and Microstructures, Institute of Semiconductors, Chinese Academy of Sciences, Beijing 100083, China; ‡ Center for Device Thermography and Reliability (CDTR), H. H. Wills Physics Laboratory, University of Bristol, Tyndall Avenue, Bristol BS8 1TL, U.K.; ¶ University of Science and Technology of China, Hefei 230026, China; # School of Science, Harbin Institute of Technology, Shenzhen 518055, China; § State Key Laboratory of Solidification Processing, School of Materials Science, Northwestern Polytechnical University, Xi’an, 710072, China; ∥ School of Advanced Materials and Nanotechnology, Key Laboratory of Wide Band-Gap Semiconductor Materials and Devices, Xidian University, Xi’an, 710071, China; ⊥ School for Engineering of Matter, Transport and Energy, Arizona State University, Tempe, Arizona, AZ85287, United States

## Abstract

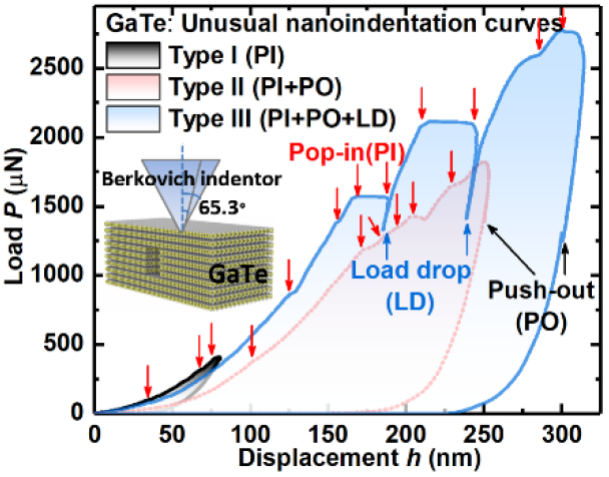

The deformation and fracture mechanism of two-dimensional (2D) materials are still unclear and not thoroughly investigated. Given this, mechanical properties and mechanisms are explored on example of gallium telluride (GaTe), a promising 2D semiconductor with an ultrahigh photoresponsivity and a high flexibility. Hereby, the mechanical properties of both substrate-supported and suspended GaTe multilayers were investigated through Berkovich-tip nanoindentation instead of the commonly used AFM-based nanoindentation method. An unusual concurrence of multiple pop-in and load-drop events in loading curve was observed. Theoretical calculations unveiled this concurrence originating from the interlayer-sliding mediated layers-by-layers fracture mechanism in GaTe multilayers. The van der Waals force dominated interlayer interactions between GaTe and substrates was revealed much stronger than that between GaTe interlayers, resulting in the easy sliding and fracture of multilayers within GaTe. This work introduces new insights into the deformation and fracture of GaTe and other 2D materials in flexible electronics applications.

Two-dimensional (2D) materials have attracted tremendous interest attributed to their extraordinary electronic, optical and mechanical properties compared to their bulk counterparts. Recently, the preparations of 2D materials through mechanical exfoliation or chemical synthesis have achieved great advances, enabling renewed investigation into 2D materials beyond graphene.^1^ Various unique optical and electrical properties have been demonstrated by 2D materials,^2−13^ such as high electron and hole mobility (2300 and 1000 cm^2^ v^–1^ s^–1^ for μ_e_ and μ_h_, respectively) in multilayered black phosphorus (BP),^14^ excellent room temperature current on/off ratio (10^8^) in monolayer molybdenum disulfide (MoS_2_) transistors,^15^ ultrahigh photoresponsivity (2 × 10^6^A/W) in gallium telluride (GaTe) multilayers.^16,17^ In addition, the mechanical properties of 2D materials are also noted to be crucial for realizing their applications in, for example, flexible, wearable and smart electronics, and have attracted much research interests. Extremely high intrinsic in-plane Young’s modulus (∼1TPa) and strength (∼130 GPa) were revealed in graphene using atomic force microscopy (AFM)-based nanoindentation.^18^ This AFM-based nanoindentation method combined with molecular dynamics (MD) and other density functional theory (DFT) calculations has been extended to measure the nanomechanical properties of other 2D materials, such as MoS_2_ and hexagonal boron nitride (hBN).^19−21^ Specifically, detailed knowledge about deformation, fracture, generation of defects and potential phase-transition of these 2D materials at nanoscale can be quite different and is essentially required when their devices are under large strain/stress or undergo numerous loading cycles.^20,22^ For example, the strength of graphene can be significantly influenced by out-of-plane deformation under external shear loading^23^ or intrinsic topological defects,^24^ leading to a failure strength of >50% reduction.

To characterize the local nanomechanical properties of 2D materials (e.g., in-plane Young’s modulus, tensile strength, and failure-strain), the aforementioned AFM-based nanoindentation is commonly used.^18,25−29^ However, inaccuracies are noted in this method, mainly due to the fact that the locally concentrated stress near the sharp AFM-tip after crack initiation cannot provide sufficient driving force for further crack propagation.^30^ Recently, full-scale nanoindentation techniques with a Berkovich-indenter have been used to measure the mechanical properties and nanometer-scale structural changes in small-volumes of materials and ultrathin-films.^31,32^ In the load–displacement curves produced by this Berkovich-tip nanoindentation method, step-like pop-in (PI) is often observed, which can be ascribed to dislocation-nucleation, slippage, phase-transition, crack-formation, and so on.^32−37^ Discontinuous-like push-out (PO) events in the unloading curve are usually reported as indications of phase-transition.^38,39^ Load-drop (LD) events (sudden load decrease at a certain displacement) can also be seen, which have been primarily correlated to the formation of interfacial cracks.^36^

When comparing the mechanical properties of reported 2D materials, interlayer interactions are noted playing an important role due to their different strength of van der Waals (vdW) forces existed between individual layers. For example, a 30% decrease in strength is observed in graphene when the number of layers increases from 1- to 8-layers;^26^ whereas the strength of hBN is insensitive to layer numbers due to its stronger interlayer vdW-interactions than that of graphene.^26^ Interlayer interactions are also found can lead to recoverable sliding within graphene multilayers during the nanoindentation loading.^28^ However, the critical mechanical properties and effects of interlayer interactions still have not been investigated in many other 2D materials including the aforementioned GaTe. Specifically, as an exciting emerging material, GaTe multilayers have demonstrated huge advantages with an ultrahigh photoresponsivity for high-performance photodetectors,^16,17^ as well as giant potential for desirable optoelectronics, electronics, and nanoelectromechanical system devices with a reported highest anisotropic resistance and a tremendous current on/off ratio within the 2D materials family;^5,40−43^ these highlighted characteristics make GaTe a great candidate for future nano- and flexible- optoelectronics, especially when its ambient degradation now can be effectively suppressed.^44^ GaTe multilayers are also distinguished by an unusually high failure-strain (an AFM-based nanoindentation measured failure-strain of 7%, which is comparable or even better than that of the commonly used PDMS or polyimide flexible substrate).^29^ Nevertheless, knowledge about the detailed deformation and fracture behaviors in GaTe multilayers which is crucial for many practical device applications is still lacking.

In this work, the nanomechanical properties of both substrate-supported and suspended GaTe multilayers are systematically characterized by full-scale Berkovich-tip nanoindentation, micro-Raman spectroscopy, AFM, scanning electron microscopy (SEM) and MD simulations. An unusual concurrence of multiple PIs accompanied by LDs events in loading curves is first observed in 2D materials family, and the mechanisms of interlayer-sliding and layers-by-layers fracture are unveiled and investigated in detail.

The GaTe multilayers samples tested are listed in Table 1 including the sample thickness, number of layers and the maximum indentation depth. It was found that the load–displacement (*P*–*h*) curves obtained from different indentation loadings performed on SiO_2_/Si-substrate-supported samples can be classified into three types; a typical curve from each type is shown in Figure 1 based on the results from different samples. The nanoindentation schematic and the GaTe atomic structure are shown in insets of Figure 1. Type-I curves are common for those at lower displacement (∼80 nm, sample 1) nanoindentations. This type of curves has only PIs; these curves are relatively smooth with multiple small PI events present where the load remained relatively constant but suddenly increases in displacement (Figure 1a). Although very small, these PIs decrease the slope of the loading curves indicating they are associated with plastic-deformation events. Type II curves are characteristic for larger displacements (∼250 nm, sample 2), and they have both PIs and POs. Some PIs associated with larger steps at higher load (>1200 μN), resulted in more pronounced slope changes in the loading curve. Further, a PO appeared in the unloading curve at ∼1300 μN, Figure 1b. Type-III curves are representative of indents collected at higher displacement (∼300 nm, sample-3) and are even more complex with PIs, LDs and POs all presented. As shown in Figure 1c, significant LDs accompanied by large PI events appear during loading, for example, LDs at (185 nm, ∼1550 μN) and (240 nm, ∼2100 μN), and a PO during unloading again at ∼1300 μN—similar to the PO observed in Type II curve in sample 2 (Figure 1b). A PO is often seen as an indicator of a phase-transition while a PI is normally correlated with dislocation-nucleation and slip behavior in Si.^39^

**Figure 1:**
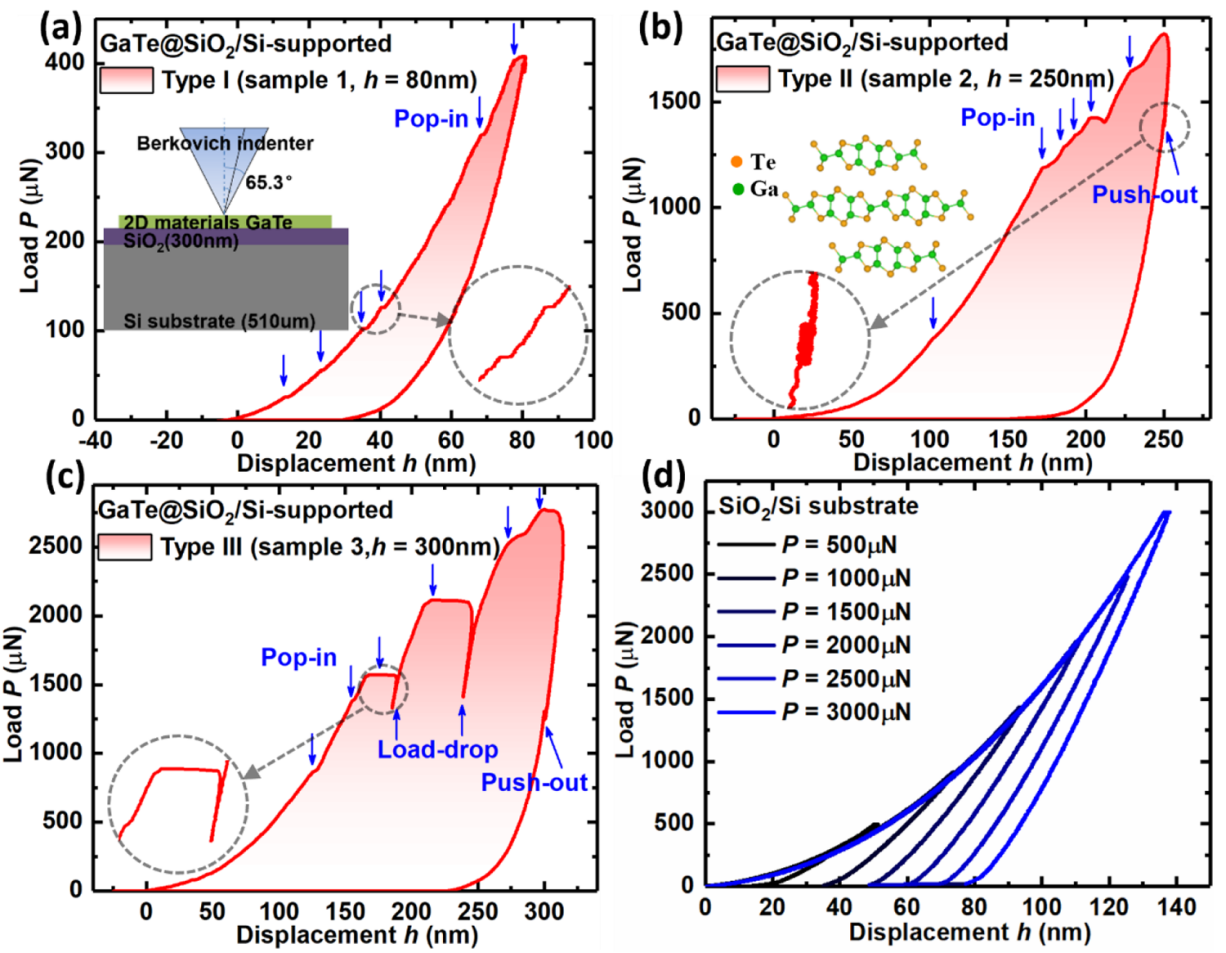
Three typical types of load–displacement (*P–h*) curves obtained on SiO_2_/Si-substrate-supported GaTe multilayers under different indentation depths: (a) Type I (sample 1, *h* = 80 nm), (b) Type II (sample 2, *h* = 250 nm), (c) Type III (sample 3, *h* = 300 nm); (d) *P*–*h* curves of the indents on SiO_2_/Si-substrate under a series of different loads. Pop-ins (PIs) and load-drops (LDs), push-outs (POs) are labeled in downward- and upward-arrows respectively in all *P*-*h* curves. Inset in panel a illustrates the schematic of the nanoindentation performed on SiO_2_/Si-substrate-supported GaTe samples; inset in panel b illustrates the *C*2*/m*-phase atomic structure of GaTe multilayers; circular insets in panels a–c are enlarged views of the representative PI, PO, and LD details.

To examine contributions from the SiO_2_/Si-substrate itself under the above testing conditions, a series of repetitive indents with increasing peak load were undertaken (Figure 1d). It shows that *P–h* curves obtained from the SiO_2_/Si-substrate are free of PIs, LDs, and POs. This confirms that the multiple PIs, LDs, and POs in *P*–*h* curves (Figure 1a–c) are genuine reflections of the mechanical responses of the GaTe multilayers. As the nanoindentation depth increases (Figure 1), the nonlinear loading curve at high-loads/displacements and the increased residual-displacement at complete unloading indicates a change from elastic to inelastic regime accompanied by increasingly pronounced energy dissipating events, such as PIs and LDs.

The above indents were compared across different indent-depths; further indents were performed on supported samples at a fixed displacement depth of 200 nm in random locations and are detailed in Figures S1 and 2. All different types of scenarios described in Figure 1 (small PIs at lower load, large PIs and LDs at higher load and POs during unloading) all repeatedly show up. Despite similar or different film thickness, some PIs (LDs) seem to appear consistently at similar depths/loads. For example, many significant PIs in the 250 nm sample (sample 2) are induced at a depth or load that similarly triggers large PIs or LDs in the 300 nm sample (sample 3), that is, about 170, 185, 210, and 240 nm in depth or 1400 and 1550 μN in load, respectively (Figure S1). This implies a sample thickness independent *P–h* curves types and a common material property driven mechanical mechanism exists in GaTe multilayers. Comparing Type III curves (Figure S1), the regularly occurring large LDs indicate multiple fracturing or cracking events in the materials during loading which suggests a transition to irreversible inelastic deformation. Large LDs at lower load, for example, ∼500 μN in sample 5, resulted in a much lower gradient of the loading curve which is a strong implication of modified compliance of the material. We also note that the observed large LDs are spaced at regular loading steps of ∼400–700 μN, and the accompanied large PI-stage lengths are spaced by ∼10–30 nm, respectively (Figures S1 and 2). This fact implies multiple step-by-step fracture and sliding behaviors are likely triggered within the GaTe multilayers, which are associated with material properties and require further analysis.

The morphology and microstructure of the indents was investigated by AFM and SEM for further understanding the different types of *P*–*h* curves. As seen from Figure 2a and b, only very small residual-imprints are left after an indentation of 80 nm, and the size of the imprints increases with increasing indentation depth. At deeper indentations up to 250 nm (Figure 2c and d), significant upward deformation of the materials was observed similar to pile-ups geometries caused by plastic-flow in ductile materials. The nature of the upward deformation observed in this work may be different, but we also call it “pile-ups” in the following. AFM measurements revealed that the heights of the three pile-ups between the sharp corners of the pyramidal indent are ∼150, 125, and 100 nm, respectively. Three cracks propagating from the corners were observed with a similar length of ∼2 μm. Notably, one of these cracks was deflected and followed a path parallel or perpendicular to the marked layer-boundaries, indicating that a favored in-plane crack direction probably exists (Figure 2c marked layer-boundary is along the armchair- or zigzag-direction^5,45^). For the indent produced by an indentation depth of 300 nm (Figure 2e and f, sample 3), more severe fractures occurred, accompanied by three asymmetric pile-ups whose heights are about 300, 50, and 50 nm, respectively. These pile-ups formed between the three corners of the indent. Again there is a favored crack direction perpendicular to the marked layer-boundary direction^5,45^ (Figure 2e). Note there is no crack in the direction parallel to the layer-boundary (both planes that parallel and perpendicular to the layer-boundary are preferred in-plane cleavage-planes^45^). These preferred crack propagation orientations indicate the anisotropic nature of the in-plane mechanical properties of GaTe multilayers.

**Figure 2:**
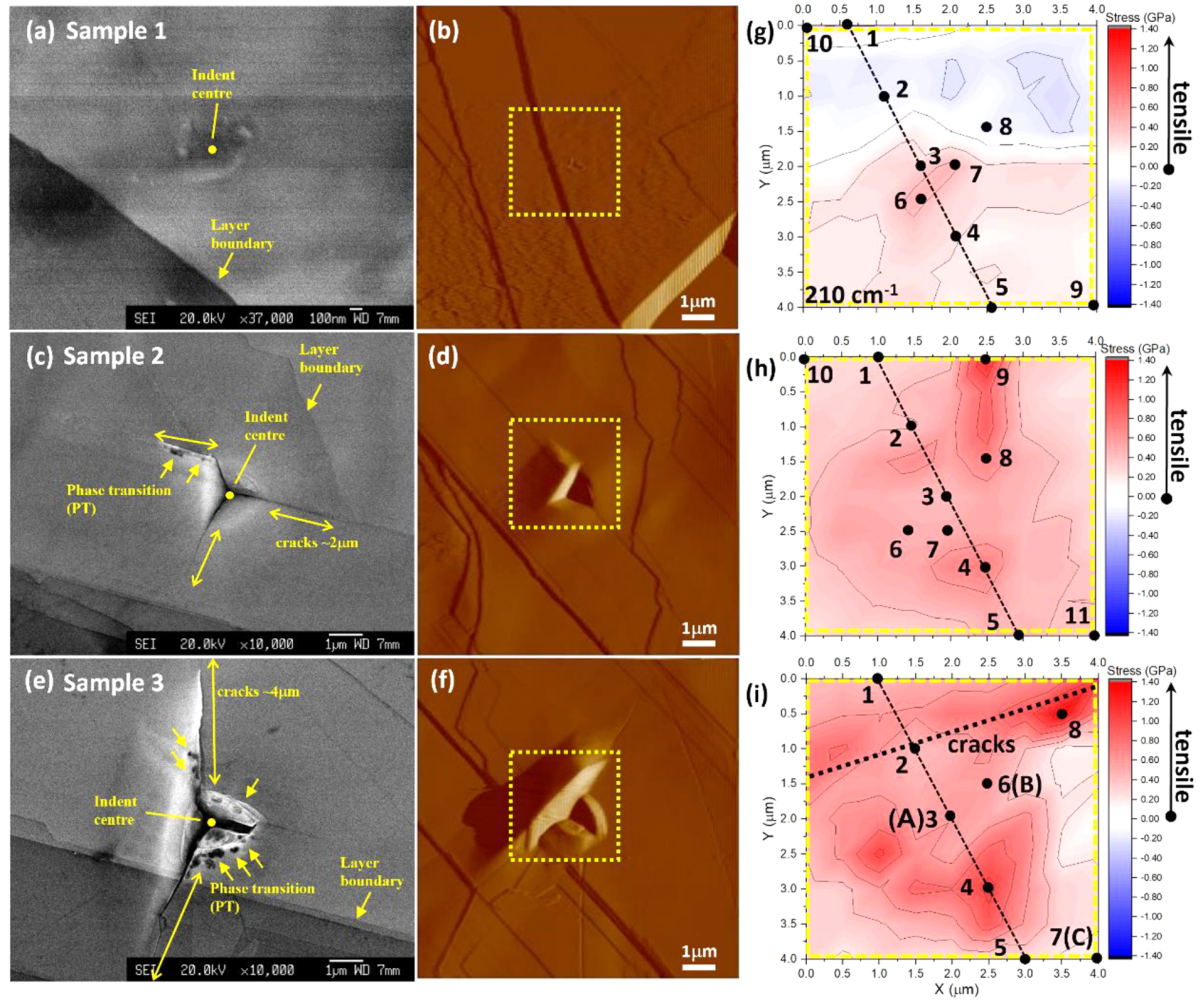
SEM and AFM images of the indent morphology for indentation depth of (a, b) 80 (sample 1), (c, d) 250 (sample 2), and (e, f) 300 nm (sample 3), with the crack lengths, layer-boundaries, indent-center, and phase-transition (PT) like dark areas being labeled. The length of the two long cracks in sample 3 reached about 4 μm. Stress mapping of the labeled open dot square area in AFM images after (g) 80 (sample 1), (h) 250 (sample 2), and (i) 300 nm (sample 3) depth indentation. The mapping area was 4 × 4 μm^2^, with a step resolution of 0.5 μm for all samples. The stress is calculated from the Raman shifts to the reference spectrum based on the stress-sensitive out-of-plane A_g_ mode (210 cm^–1^) using an experimentally obtained stress coefficient of 2.59 cm^–1^/GPa; the error bar represents the homogeneity of stress distribution. The dash black line is along one of the axis of the pyramidal indent area, and the black dots are selected and numbered for the following Raman spectra comparison (see Figure S4).

SEM images of other indents performed under the same displacement depth were further analyzed and summarized in Figure S3. Similar indent morphologies as that in Figure 2 are observed. On the basis of the morphology features and the *P*–*h* curves (Figures 1, 2, S2, and S3), the following correlation can be summarized: (i) Pyramidal indent-imprints without macro-cracks, fracture, or pile-ups around the indent result in the Type I *P*–*h* curve. As there is no fracture or cracks in the residual-imprints (Figure 2a), the small PIs at low-load (<500 μN) in Figure 1a can be correlated to plastic-deformation mechanisms other than fracture and cracks. (ii) Pyramidal indent-imprints with three similar sizes of pile-ups of surrounding materials and macro-cracks lead to Type II *P*–*h* curves. More pronounced PIs are caused by the formation of macro-cracks during the indentation loading process, and this resulted in larger permanent residual-displacement upon the reaction of the load. (iii) Pyramidal indent-imprints with fracture, asymmetric pile-ups of the surrounding materials and crack propagations can be correlated to the Type III *P*–*h* curve. In this case, the events of larger PIs and significant LDs can be attributed to the formation of obvious fracture in the indent-center. It is worth noting that, in the residual-indents, areas with different contrast (Figure 2c–e) are consistently observed; these dark areas are likely results of phase-transition of materials driven by the local high-stress generated underneath the indenter.

To further understand the stress distribution after the deformation and fracture process, micro-Raman spectroscopy was used to evaluate and map the residual-stress distribution around the indents. From the indent-imprint created at 80, 250, and 300 nm-depth, the residual-stress maps (Figure 2g–i) showed an average tensile-stress of about 0.07 ± 0.18, 0.42 ± 0.16, and 0.51 ± 0.25 GPa, respectively. For the residual-imprint from the 300 nm-depth-indent (sample 3, Figure 2i), the average residual-stresses formed along the cracks and the edges of the indent-imprints are significantly larger (∼0.9 GPa) than that away from the cracks (∼0.1 GPa). The pile-up region exhibits a higher maximum tensile-stress (∼1.3 GPa) than that of the indent-center (∼0.5 GPa), but both regions have much higher stress than that of areas away from the indents and cracks (∼0.1 GPa). Also, there are large variations of residual-stresses over the imprint area, Figure 2i. For the residual-imprints of the 80 nm (sample 1) and 250 nm depth indents (sample 2), a more homogeneous tensile-stress distribution was present around the indent-center, Figure 2g–h. The highest tensile-stress in the 250 nm-imprint (sample 2) is ∼1.0 GPa and located in the pile-up region ahead of the macro-crack, similar to the localized stress concentration at the crack-tip in 300 nm-imprint (sample 3, Figure 2i). For the residual-stress obtained on the same 200 nm-depth-indents (samples 4 and 5, Figure S4), similar average stress of ∼0.2 GPa and largest tension of ∼0.7 GPa appear in both Type II and Type III indents. To conclude, the average residual-stress around the residual-imprints increased with the indentation depth. In areas away from the indents and cracks, a small tensile-stress of ∼0.1 GPa exists for all samples. Larger tension is normally found in the pile-up region and the corners of cracks. The indentation process has introduced deformation to the material and as such large localized tensile-stress was formed. This has subsequently resulted in the formation of cracks along the weaker directions. Continued indentation to a higher depth acted as a crack driving force to promote more crack growth, and this is evidenced by the higher tensile-stress at the crack tips (Figure 2h and i).

Micro-Raman spectra along one axis of triangular imprint from 300 nm-indent (sample 3, Figure 2i) were inspected in more detail in Figure 3a. A significant difference is evident between the indent-center (A), the pile-up region (B), and the area away from the indent and cracks (C). Specifically, broadened Raman peaks associated with amorphization/disorder are observed in the spectrum collected at point B. Most importantly, several new peaks (e.g., 89, 118 cm^–1^) appeared while some originally existing peaks became less apparent (109, 114, 126, 143, 209, and 282 cm^–1^) or even disappeared (75, 162, 176, 268 cm^–1^) in the imprint region. Similarly, for the 80 (sample 1), 200 (samples 4 and 5), and 250 nm (sample 2) indent-imprints, new peaks around 90 and 99 cm^–1^ are also apparent in the near-indent-center region (Figures S5 and S6); but no significant peak broadening as that of point B was observed.

**Figure 3:**
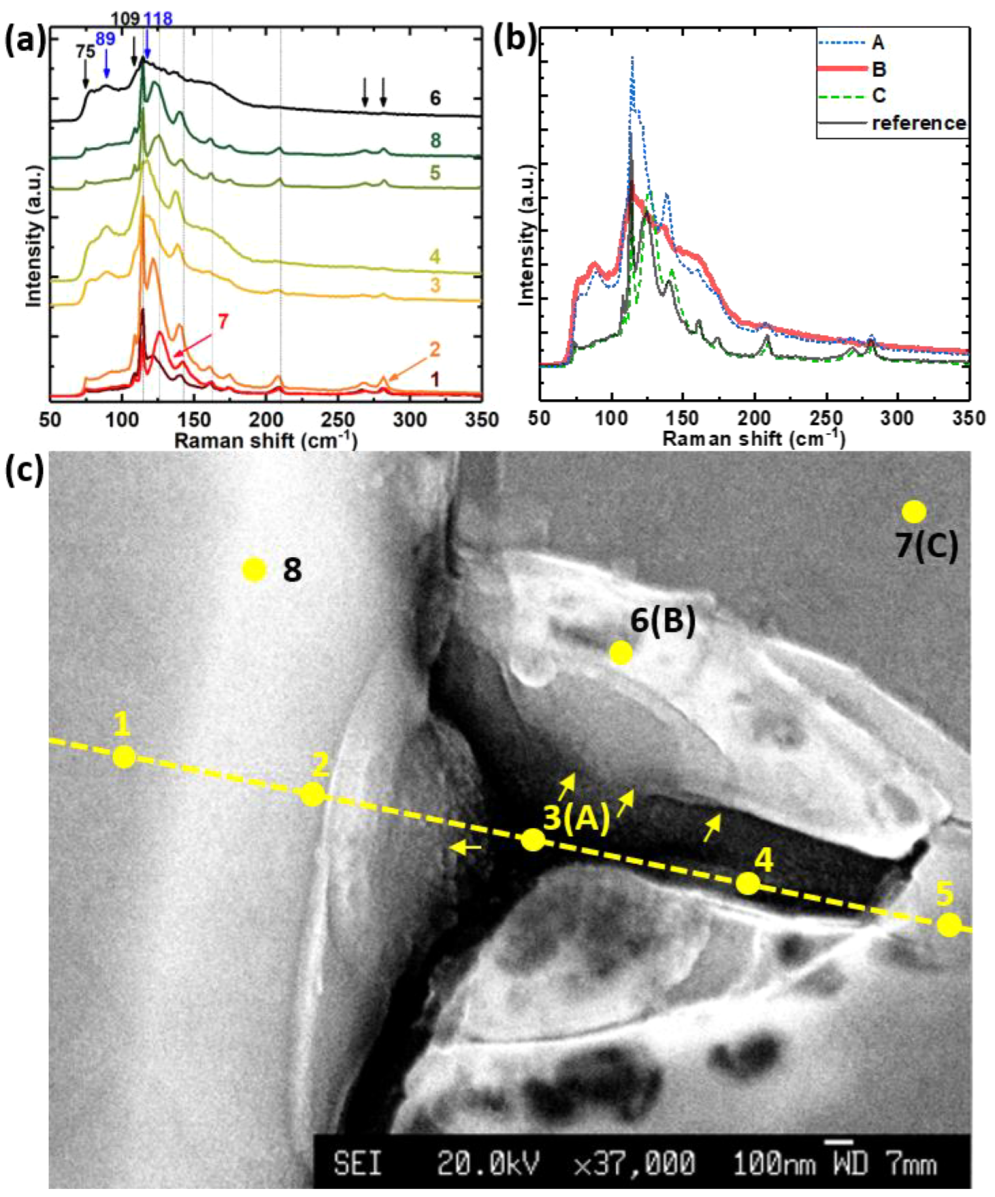
GaTe multilayers after 300 nm-depth (sample 3) indentation: (a) micro-Raman spectra evolution along one axis of the pyramidal indent as the selected points marked in Figure 2i; (b) detailed Raman spectra comparison between the indent-center and nonindent area, where A, B, and C are marked in Figure 2i. The reference spectrum was collected before the nanoindentation. (c) SEM image of the detailed morphology of indent-center for the sample under 300 nm-depth (sample 3) nanoindentation, with the arrows indicating amorphous-like phase-transition areas as evident in the Raman spectra.

Raman spectra with sharp peaks obtained at the indent-center (point A) after 300 nm-depth-indent (sample 3) indicates the amorphization or disorder transition is only localized within the fractured pile-up area; combined with the appearance of multiple LDs, this further implies that the GaTe layers are likely partially fractured layer-by-layer and slide away within the interlayers. We do not exclude the possibility of the formation of new phases during the nanoindentation process induced by the large local stress. Indeed, the areas with darker contrast in the SEM micrographs (Figures 2c–e and 3c) may originate from changed electrical conductivity in the amorphous-like or new phased transformation materials, similar to those reported in 4H-SiC^46^ and GaAs.^47^ Notably, the Raman spectra of indent center and darker areas (e.g., points 3, 4, and 6) comprise features of both original phase and transformed phase, further indicating these regions are composed of mixed-phases rather than single-phase only.

To better understand the nanoscale events associated with the indention process, MD simulations were performed for SiO_2_/Si-substrate-supported GaTe multilayers. Both defect-free and defected GaTe were considered, with the configuration of the model shown in Figure 4a. The simulated load-depth curves from three types of thin samples are overlaid in Figure 4b. As evident for all three samples, there is a linear-elastic region up to 10 nN before LDs occurred and lead the deformation to the inelastic or plastic regime. This is consistent with experimental data. At higher loads, the magnitude of the LDs become more pronounced. Differences to the experiment are due the finite number of layers which can be considered in MD modeling limited by the calculation efficiency and resources, but the main qualitative features from the experiments are reflected in the simulated curves, that is, concurrence of multiple PIs, LDs, and POs. As clearly displayed in Figures 4c–f and S9, various levels of interlayer-sliding along *x*-direction accompanied by fracture at different indentation depths are obvious. Therefore, it is apparent from the MD simulations that the original mechanical mechanism of these concurrence events is correlated to layer-by-layer fracture and interlayer-sliding.

**Figure 4:**
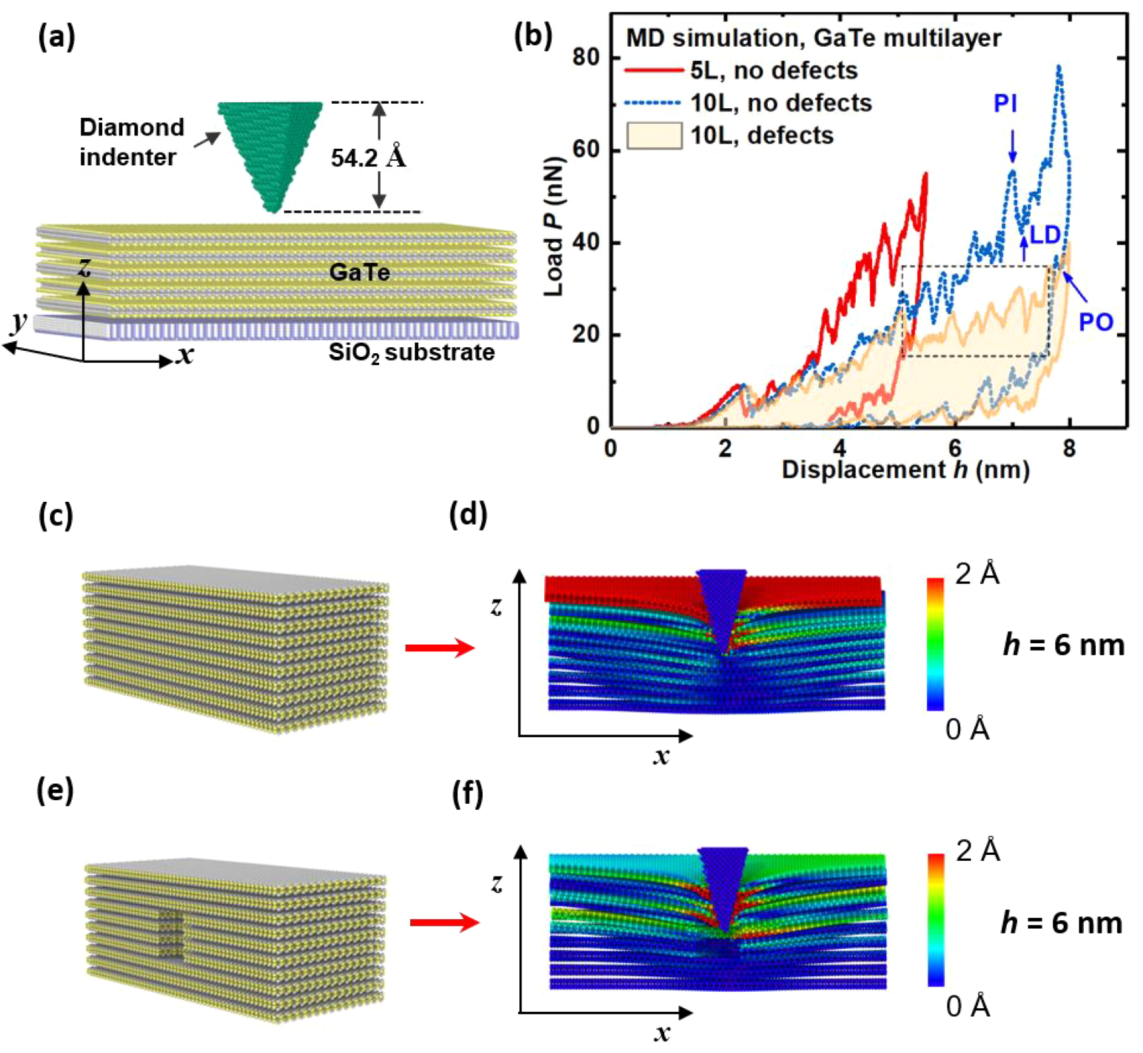
(a) Sample structure model with a 5-layer GaTe sample of simulated nanoindentation performed by molecular dynamics (MD). (b) Comparison of MD simulated nanoindentation curves for 5-layers (5L) and 10-layers (10L) samples with and without the existence of void-defects. Upward and downward arrows represent LDs (PO) and PIs, respectively. (c) Sample structure model for “10L, no defects” in MD simulations. (d) Fracture and interlayer sliding along *x*-direction at *h* = 6 nm for “10L, no defects” obtained in MD simulations. (e) Sample structure model for “10L, defects” in MD simulations. (f) Fracture and interlayer-sliding along *x*-direction at *h* = 6 nm for “10L, defects” obtained in MD simulations. The colormap represents the different quantity of the atomic displacements during loading process at the indent-depth of 6 nm.

Furthermore, it was found that compared with the 10-layer-without-defects sample, the introduction of inevitable void-clusters defects reduced the loading slope after ∼5 nm-depth and 20 nN-load (Figure 4b). This is consistent with the “softening” of the loading curve observed in the experiments (e.g., PI-stages and LDs in Figure 1b and c). This also implies the larger PIs and LDs are likely to originate from the multiple layer-to-layer cofracture and cosliding within GaTe multilayers facilitated by defects/defects-clusters. For the same indentation depth, the maximum load reached in the “defected” 10-layer sample (∼40 nN) has reduced to ∼50% of the load in the “defect-free” 10-layer sample (∼80 nN). The events of PIs, LDs, and POs are all preserved, but their magnitude is changed. In the 10-layer-with-defects samples, fracture across multiple layers have been observed and more significant internal interlayer-sliding is present (Figures 4e–f and S9), corresponding to the relative larger PI-stages in the experiments. Moreover, the simulations also unveil that the vdW-interaction-force between GaTe multilayers and the substrates (1.83 eV/nm^2^) is much stronger than that between GaTe interlayers (0.56 eV/nm^2^), thus resulting easier fracture of GaTe layers and interlayer-sliding instead of slippage on the substrate.

To provide further evidence for the above mechanical mechanism and exclude any influence from the substrate, nanoindentation experiments were performed on GaTe multilayer samples suspended on rectangular membrane slits fabricated on the SiO_2_/Si-substrates. Indentation was used to deform the 2D GaTe sample in bending up to 250 nm (Figure 5). The concurrence of multiple small PIs and LDs, instead of smooth elastic behavior including that at low-load, is observed for all tests (Figure 5b). These multiple small PIs events are similar to the behavior reported in single-crystal Pt,^48^ that is, a transition from elastic- to plastic-deformation because of dislocations nucleated under the indenter-tip. Multiple small LDs following some PIs are also evident including at depths of ∼120, ∼180, and ∼240 nm, which agree well with those LDs observed in supported GaTe samples (Figure 1).

**Figure 5:**
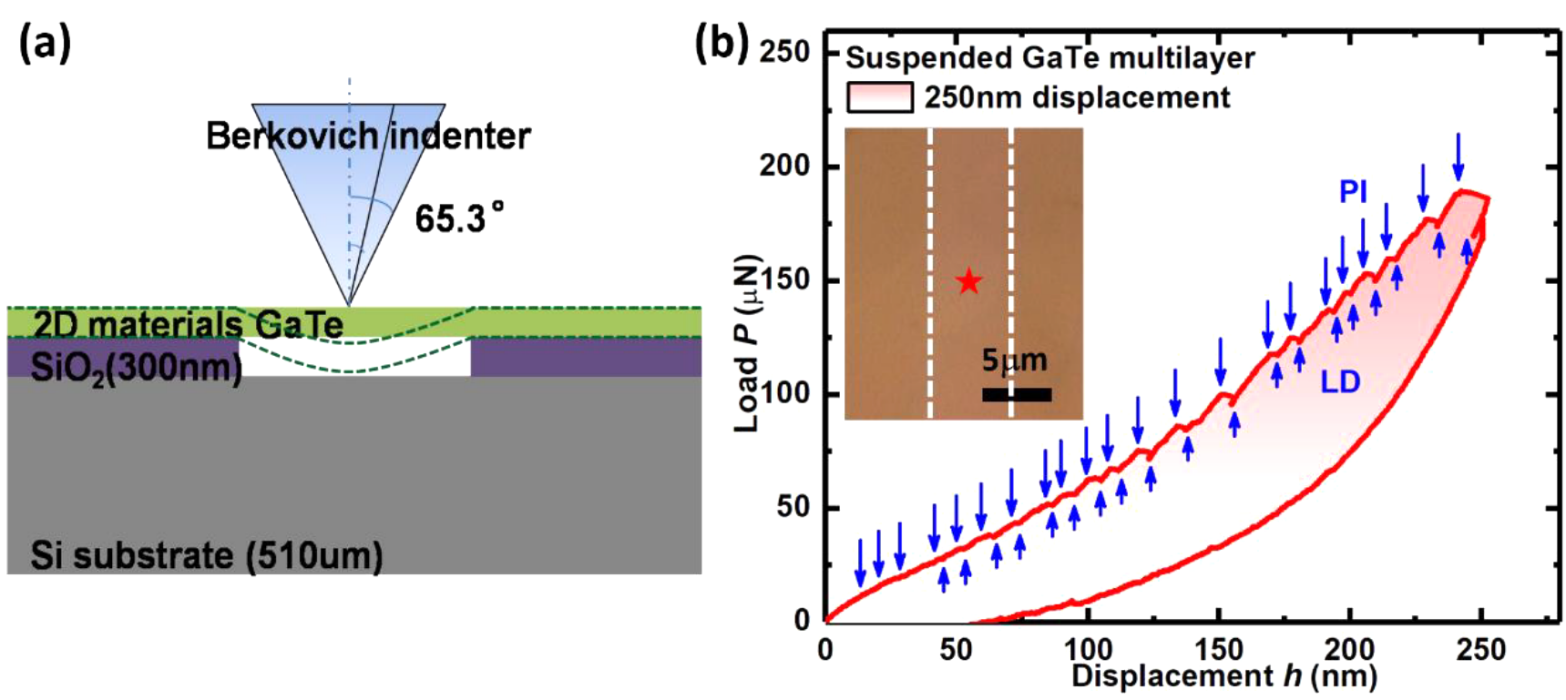
(a) Schematic of nanoindentation experiment performed on suspended GaTe samples. (b) *P*–*h* curve under 250 nm depth (sample 7) indentation for suspended GaTe multilayers. The slits width is ∼6 μm, and is larger than the cross-sectional tip-radius of ∼1087 nm at the depth of 250 nm (calculated from *R* = 2*h*_p_ tan 65.3°, where *h*_p_ is the indent-depth and *R* is the cross-sectional tip-radius at *h*_p_). Upward and downward arrows represent LDs and PIs, respectively. Inset: Optical microscopy image for one GaTe multilayer sample suspended on a membrane slit in the supporting SiO_2_/Si-substrate; the mark represents the position where nanoindentation was performed.

However, it is worth noting that no detectable fracture/cracks or a permanent-set indent-imprint could be observed (either by AFM or SEM) even after nanoindentation to 250 nm (the maximum available indentation depth in this sample, Figure S8). This indicates that the multiple small PIs and LDs are inner features generated within the GaTe multilayers, instead of surface fractures. As the sample was suspended, the maximum load (180 μN) was much lower than that of supported samples (1800 μN) under the same 250 nm displacement. This is plausible as the suspended GaTe films are subjected to mainly bending while the supported films were in more compression as they are supported by a substrate. The absence of large LDs in suspended GaTe films suggests again that those LDs observed in supported films are correlated to the macro-cracks and fractures on the surface.

Performing micro-Raman spectra mapping, similar to Figures 2 and 3, we excluded possible structural changes and stress variations induced during the nanoindentation around the indent-imprints in the suspended films. The measured Raman spectra show no frequency change (Figure S8), indicating a less-degraded crystal quality on the sample surface after the nanoindentation than that for the fully supported materials. The Raman spectra evolution along both vertical and horizontal directions of the indent-center shows no other significant difference. This result not only validates the good quality of the crystals in the suspended GaTe after nanoindentation, but also indicates that those PIs and LDs are not attributed to phase-transition or crystal structure changes. As revealed by MD simulations, concurrence of multiple small PIs and LDs in suspended GaTe multilayers are also ascribed to the layer-by-layer fracture, and step-to-step interlayer-sliding behaviors generated within the GaTe multilayers driven by the lateral force from the indenter. Some PIs and LDs are also noted to be a bit larger which can be attributed to defects-mediation.

In summary, the deformation and fracture behaviors of both SiO_2_/Si-substrate-supported and suspended GaTe multilayers were investigated through full-scale Berkovich-tip nanoindentation, micro-Raman spectroscopy, AFM, SEM, and MD simulations. An unusual concurrence of multiple PIs and LDs events is observed in the loading curves of supported GaTe multilayers, accompanied by fracture and cracks. This phenomenon also appears in suspended GaTe multilayers, but without any observable fracture or cracks on the surface. Qualitative MD simulations reveal that such concurrence of multiple PIs and LDs in the loading curve originates from layers-by-layers cofracture and step-to-step interlayer-sliding within GaTe multilayers during nanoindentation. The vdW-interaction-force between GaTe multilayers and the substrates is also revealed much stronger than that between GaTe interlayers, thus resulting in the easier fracture and sliding of materials within the GaTe multilayers instead of slippage on the substrate. This work unveils a new deformation and fracture mechanism within multilayered 2D materials and will underpin device designs, especially for nanoflexible devices based on similar 2D layered materials.

## Supporting Information Available

Detailed description of the experimental synthesis, characterizations, and first-principles calculations and supplementary results of nanoindentation characterizations, morphology and microstructure analysis, micro-Raman spectrum and stress analysis, AFM and micro-Raman spectrum, and molecular dynamics simulations (PDF)
