# Supplementary material for: Unusual Deformation and Fracture in Gallium Telluride Multilayers
Source: J Phys Chem Lett. 2022 Apr 25;13(17):3831–9. doi: 10.1021/acs.jpclett.2c00411 (PMC9082608; doi:10.1021/acs.jpclett.2c00411)
Supplement: Supplementary file 1 — jz2c00411_si_001.pdf [file jz2c00411_si_001.pdf]

Supporting Information:

Unusual Deformation and Fracture in Gallium Telluride Multilayers

Yan Zhou[†,‡,#], Shi Zhou[¶,#], Penghua Ying[※], Qinghua Zhao[§], Yong Xie[‖,*], Mingming Gong[§], Pisu Jiang[‡], Hui Cai[⊥], Bin Chen[⊥], Sefaattin Tongay[⊥], Jin Zhang[※,*], Wanqi Jie[§], Tao Wang[§,*] Pingheng Tan[†], Dong Liu[‡], and Martin Kuball[‡,*]

[†] *State Key Laboratory of Superlattices and Microstructures, Institute of Semiconductors, Chinese Academy of Sciences, Beijing 100083, China.*

[‡] *Center for Device Thermography and Reliability (CDTR), H. H. Wills Physics Laboratory, University of Bristol, Tyndall Avenue, Bristol BS8 1TL, UK.*

[¶] *University of Science and Technology of China, Hefei 230026, China.*

[※] *School of Science, Harbin Institute of Technology, Shenzhen 518055, China.*

[§] *State Key Laboratory of Solidification Processing, School of Materials Science, Northwestern Polytechnical University, Xi'an, 710072, China.*

[‖] *School of Advanced Materials and Nanotechnology, Key Laboratory of Wide Band-Gap Semiconductor Materials and Devices, Xidian University, Xi'an, 710071, China*

[⊥] *School for Engineering of Matter, Transport and Energy, Arizona State University, Tempe, Arizona, AZ85287, USA*

[#]Contributed equally, co-first author.

[*]Corresponding author. Email address: martin.kuball@bristol.ac.uk, yxie@xidian.edu.cn, taowang@nwpu.edu.cn, jinzhang@hit.edu.cn.

Keywords: Two-dimensional layered materials, gallium telluride, mechanical deformation, fracture, nanoindentation, interlayer sliding.

Table of contents:

1. Experimental details and methods

2. Supplementary nanoindentation characterization and comparison

3. Supplementary morphology and microstructure analysis of nanoindentation

4. Supplementary micro-Raman spectrum and stress analysis of nanoindentation

5. Supplementary AFM and micro-Raman spectrum of suspended samples

6. Supplementary Molecular Dynamics simulations

1. Experimental details and methods

1.1 Materials and samples preparation

The single-crystal bulk GaTe ingot was grown by the modified vertical Bridgeman method, with the help of an accelerated crucible rotation technique to improve the mass and heat transport and smoothen the solid-liquid interface during the crystal growth. High purity powders of gallium (99.99%, Alfa Aesar) and telluride (99.99%, Alfa Aesar) with chemical stoichiometry were mixed in a rocking synthesize furnace and sealed in an evacuated quartz ampoule ($<10^{-4}$ torr vacuums). GaTe flakes were mechanically exfoliated onto 300 nm SiO_2/Si or PDMS substrates from a single-crystal bulk GaTe wafer cut from the above GaTe ingot.

1.2 GaTe multilayers transfer and membrane slits fabrication

To characterize the intrinsic in-plane mechanical properties of both supported and suspended 2D GaTe, free-standing GaTe multilayers were transferred onto SiO_2/Si substrates and a series of membrane slits, which were patterned and fabricated onto SiO_2/Si substrates via standard CMOS processing, to form supported and suspended samples, respectively (Table S1). After being exfoliated from the bulk using Scotch tape, the GaTe multilayers including tape were attached to a PDMS substrate where thinner GaTe multilayers can be further exfoliated from the tape. GaTe multilayers on PDMS were characterized using micro-Raman spectroscopy at a laser excitation wavelength of 488 nm; no detectable glue residue on the GaTe multilayers was also confirmed using this method. A similar pick-up dry transfer technique[1-3] was developed to transfer the target GaTe multilayers onto designated membrane slits fabricated on the SiO_2/Si substrates. The rectangular geometry of these slits was 3-6 μm in width, 20-40 μm in length, and these slits were etched through the whole 300-nm-thick SiO_2 films which were thermal oxidized onto the surface of Si substrates.

1.3 Materials properties characterization

To investigate and understand the mechanical properties of 2D GaTe multilayers, nanoindentation tests were performed using a Hysitron TI 980 Nanoindenter on both supported and suspended GaTe multilayers. A series of indents with different indent depths controlled under the displacement mode were generated using a Berkovich indenter with a 65.3^{o} tip angle. To explore the morphology evolution and visualize the crack details of the indents that were induced by different forces, high-resolution field-emission SEM (Quanta200 FEG, FEI) images were taken. Detailed sample

thickness, indentation depths and the tomography of indents were obtained through AFM (Bruker Dimension Edge) using the tapping mode. Residual stress and its distribution after nanoindentation were characterized through the shifts of Raman modes (measured by Renishaw InVia Raman spectrometer at a laser excitation wavelength of 488 nm, using a 100×0.9NA objective with a spot radius of 0.44±0.02 μm), calibrated with the Si Raman line. A reference Raman spectrum of GaTe flakes was measured prior to nanoindentation in the indent area which was later used for the evaluation of residual stress. Raman spectra within the indent region were analyzed for indications of possible phase transformation.

Table S1. List of the selected GaTe multilayers samples plotted in the figures and their thicknesses, number of layers and maximum indent depths determined by AFM.

**Sample number**	**Indent depth, *h* (nm)**	**Sample thickness (nm)**
Sample 1 (supported)	80	301.8 (178 layers)
Sample 2 (supported)	250	354.3 (209 layers)
Sample 3 (supported)	300	372.1 (219 layers)
Sample 4 (supported)	200	321.7 (189 layers)
Sample 5 (supported)	200	373.8 (220 layers)
Sample 6 (supported)	250	779.7 (459 layers)
Sample 7 (suspended)	250	779.7 (459 layers)

1.4 Simulation Methods

MD simulations conducted in this work were implemented by using the open-source simulation code LAMMPS,[4] in which the standard Newton equations of motion were integrated over time using the velocity Verlet algorithm with a time step of 1 fs. Neglecting the presence of the Si substrate, the simulation box consists of three parts: the diamond indenter, the GaTe multilayers and the SiO_2 layer underneath the GaTe (Figure 4 in the main text). Cubic and stishovite crystal structure were adopted for the diamond indenter and the SiO_2, respectively. The GaTe structure was obtained from first-principles calculations, in which the bond length $d_{\text{Ga-Te}}$ and $d_{\text{Ga-Ga}}$ is 2.70 Å and 2.46 Å,[5] respectively. In MD simulations, 5-layered and 10-layered GaTe nanosheets were considered. The spacing of adjacent GaTe layers is 9.2 Å,

while the distance between the lowermost GaTe layer and the SiO_2 is 5 Å. These values were obtained after a sufficiently long relaxation achieving a convergence in the simulation. The initial distance between the indenter and the uppermost GaTe layer was set as 10 Å to avoid vdW interactions. During the whole simulation process, both diamond indenter and SiO_2 layer were regarded as a rigid body by setting the velocity of their atoms as zero. The force interactions between atoms in monolayer GaTe were described by the Stillinger-Weber potential using parameters from Jiang *et al.*[6] The C-C interactions in the indenter were described by the adaptive intermolecular reactive empirical bond order potential,[7] while the interactions between atoms in SiO_2 were described by the Tersoff potential[8] using parameters from Munetoh *et al.*[9] The vdW interactions between adjacent GaTe layers (Ga and Te atoms), the indenter and GaTe multilayers (Ga, Te and C atoms), the GaTe multilayers and substrate (Ga, Te, Si and O atoms) were described by the 12-6 Lennard-Jones (LJ) potential. Detailed parameters used in the LJ potential are listed in Table S2.

Periodic boundary conditions were applied along in-plane *x* and *y* directions, while free boundary condition was used along the *z* direction. Before loading, the GaTe multilayer system was relaxed at 1K in the NVT ensemble (constant atom number, volume and temperature) for 100 ps to obtain an equilibrium structure with a stable energy. After sufficient relaxation, the indenter moves down at a velocity of 0.05 Å/ps during the loading process until achieving the specified displacement or load; then, the indenter moves backwards to the initial position with the same velocity. Note that the force obtained was calculated as the total force on the centroid of the indenter.

Table S2. Lennard-Jones (LJ) potential parameters for the nanoindentation MD simulations. Arithmetic mix rule is employed to model the LJ potential between different elements.

Atom-Atom	C-C [10]	Ga-Ga [11]	Te-Te [12]	Si-Si [13]	O-O [10]
ϵ (eV)	0.00455	0.00445	0.01727	0.01740	0.00260
σ (Å)	3.4000	1.60000	3.98396	3.82600	3.15000

2. Supplementary nanoindentation characterization and comparison

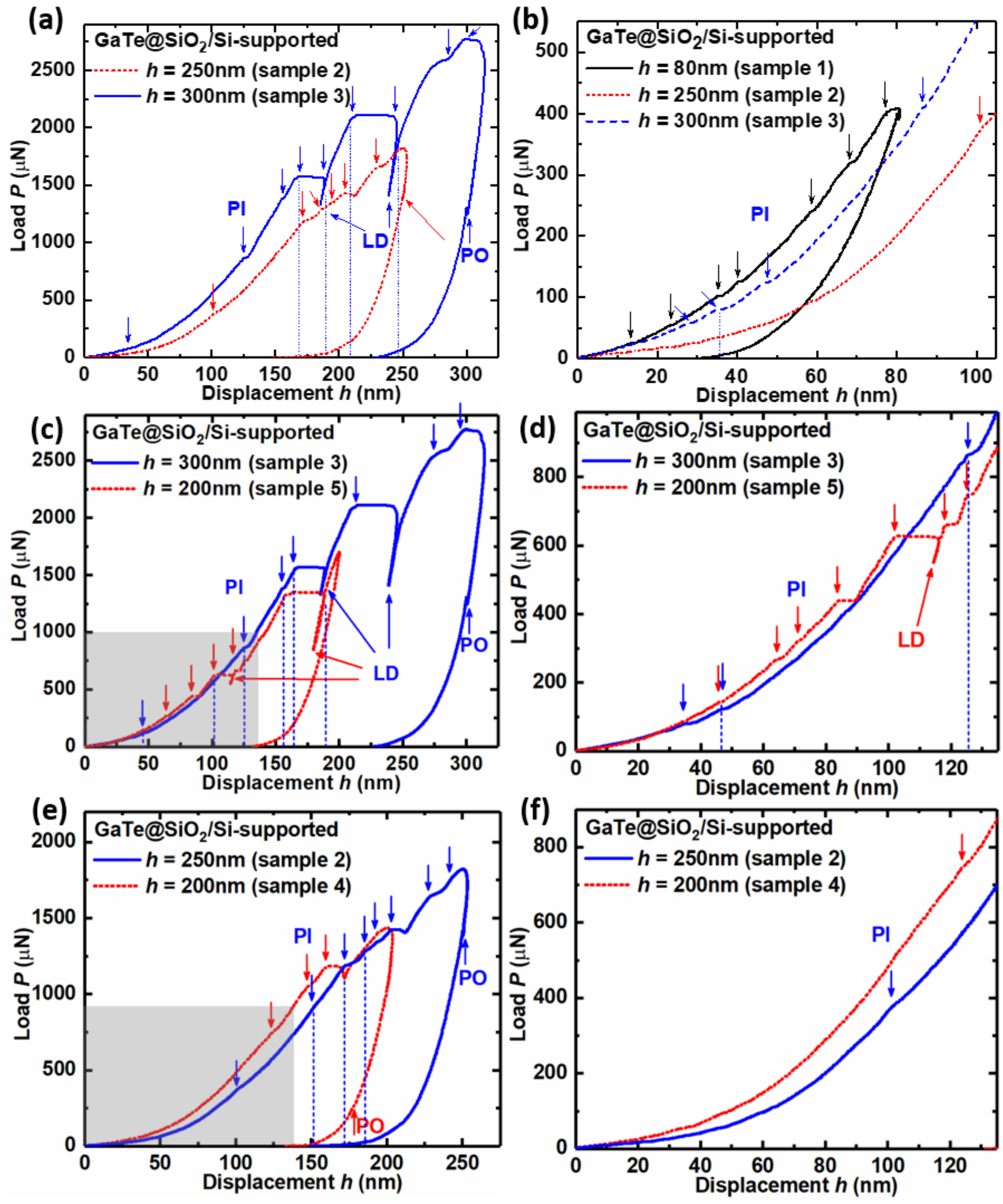

Figure S1. Comparison of *P*-*h* curves for samples: (a) at indentation depth of *h*=250nm (sample-2) and *h*=300nm (sample-3); (b) details of lower loading region (0-105nm) for *h*=80nm (sample-1), *h*=250nm (sample-2) and *h*=300nm (sample-3) samples. (c) at indentation depth of *h*=300nm (sample-3) v.s. *h*=200nm (sample-5) for Type-III, which with almost the same layer thickness (see Table 1, only one layer difference, ~1.7nm for each monolayer); (d) details of lower loading region (0-135nm) of curves in (c), shaded region; (e) at indentation depth of *h*=250nm (sample-2) v.s. *h*=200nm (sample-4) for Type-II, which with different layer thickness, and (f) details of lower loading region (0-135nm) of curves in (e), shaded region. Pop-ins (PIs) are

labeled in downward arrows while load-drops (LDs) and push-outs (POs) are labeled in upward arrows respectively in all *P-h* curves; the vertical dash lines mean PI (LD) on the compared *P-h* curves appeared at similar indentation depth.

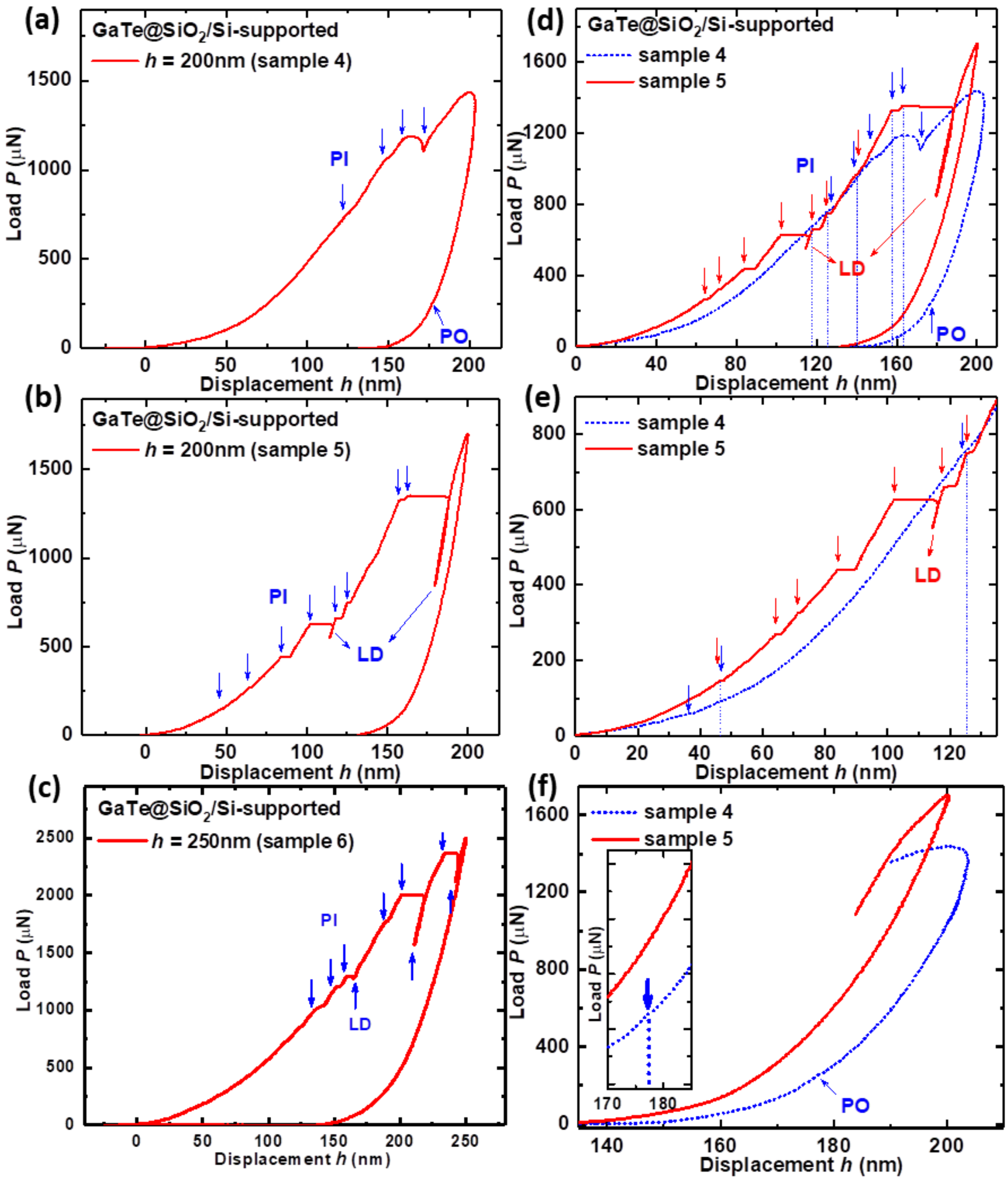

Figure S2. *P-h* curves obtained under the same indentation depth of 200nm in the displacement-controlled mode for: (a) sample-4 (Type-II), and (b) sample-5 (Type-III). (c) *P-h* curves obtained under the same indentation depth of 250nm in the displacement-controlled mode for sample-6 (Type-III). (d) Comparison of *P-h* curves for sample-4 and sample-5, with their lower loading region (<135nm) and unloading region details shown in (e) and (f), respectively. PIs and LDs (POs) are labeled in downward and upward arrows respectively in all *P-h* curves.

3. Supplementary morphology and microstructure analysis of nanoindentation

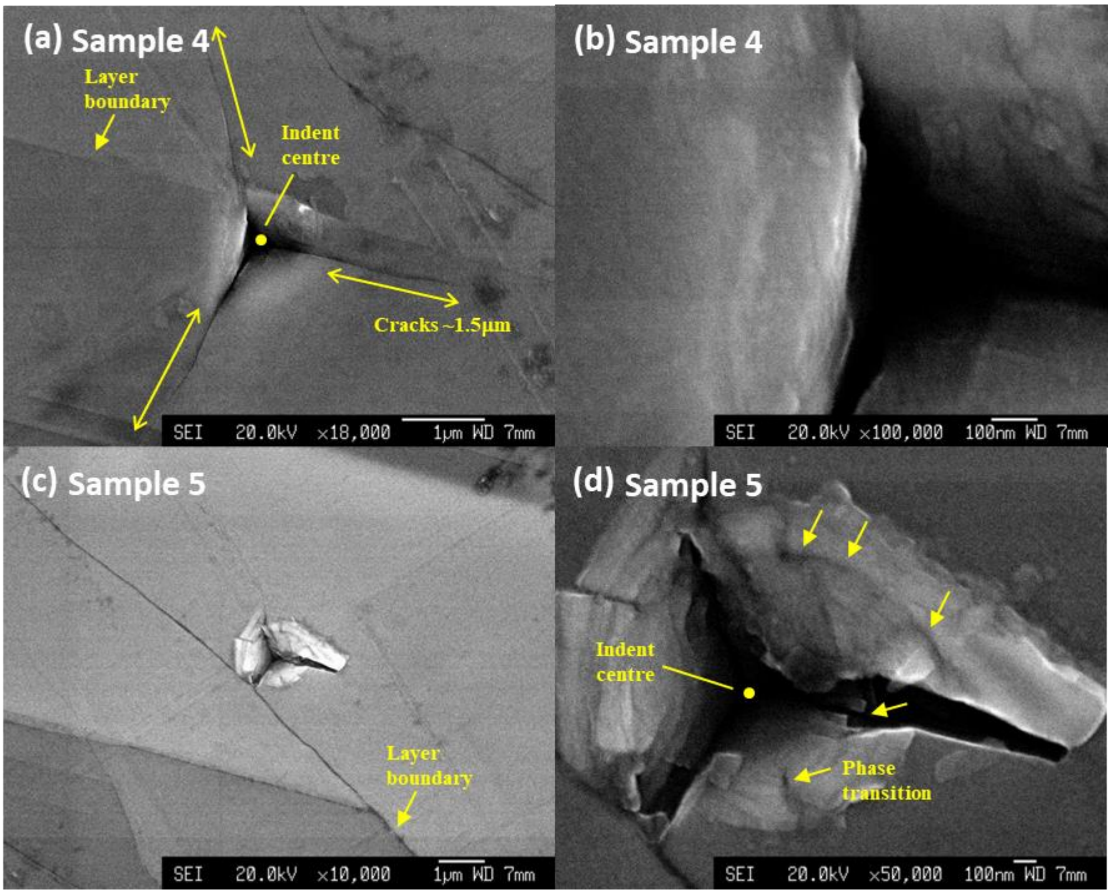

Figure S3. SEM images of the residual indent imprints under a same depth indentation of 200nm, with the cracks-lengths, indent-center, layer-boundaries and phase-transition features being labeled. Image pairs of (a, b) and (c, d) correspond to sample-4 and sample-5 shown in Figure S2(a) and (b), respectively. In sample-4, three almost symmetric pile-ups around the pyramidal imprint were observed accompanied by three similar cracks with lengths of ~1.5μm. In sample-5, the pile-ups are more pronounced but there is no obvious formation of cracks. It should also be noted that only in sample-5, some weak 'darker' fractural features of materials as those in Figure 3-4 were observed.

4. Supplementary micro-Raman spectrum and stress analysis of nanoindentation

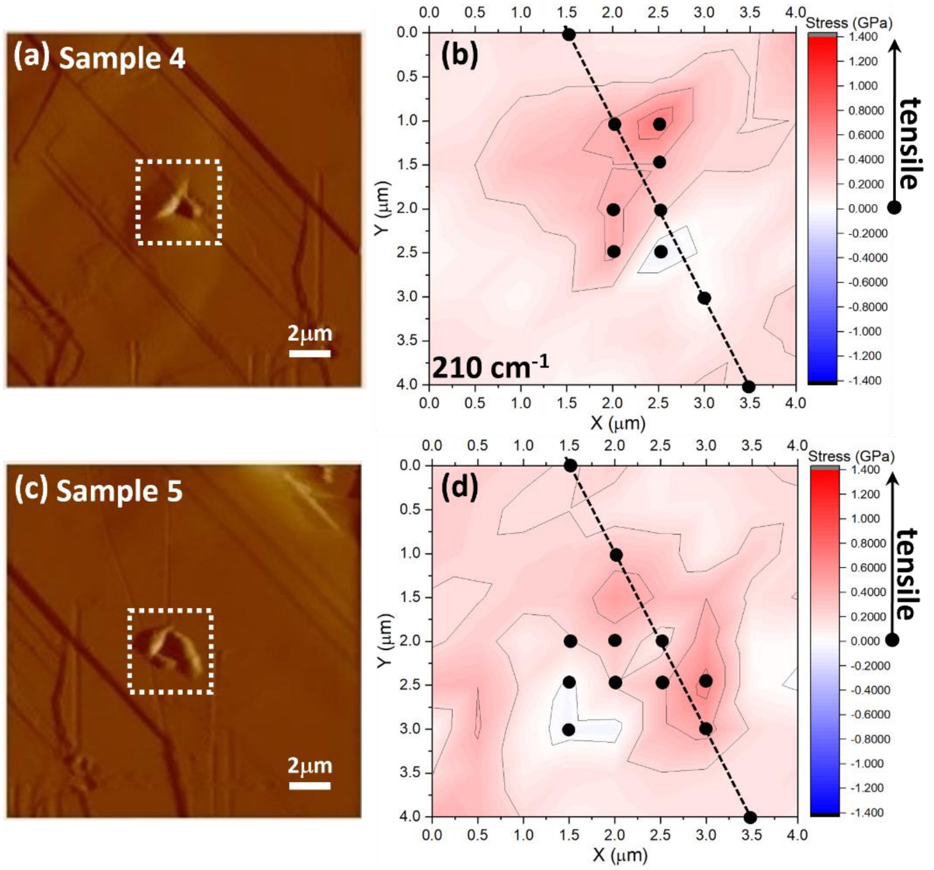

Figure S4. AFM images and Raman mapping area (4×4μm^2, indicated by open dot square) of samples after nanoindentation under 200nm-depth displacement load for: (a, c) sample-4 and sample-5, respectively. (b, d) Stress evaluation mapping of the labeled open dot square area for sample-4 and sample-5, respectively. The mapping area has a step resolution of 0.5μm for all samples. The stress is calculated from the Raman shifts to the reference spectrum based on the stress-sensitive out-of-plane A_g mode (210cm^{-1}) using an experimentally obtained stress coefficient of 2.59cm^{-1}/GPa. The black line is along one of the axis of the pyramidal indent area and the black dots are selected for Raman spectra comparison (see Figure S6).

Figure S4(b, d) shows an average residual stress of about 0.18±0.12GPa (tensile) and 0.20±0.13GPa (tensile) was created in sample-4 and sample-5 after the nanoindentation, respectively (the error bar represents the homogeneity of stress distribution). It is also shown that fracture tends to result in larger tensile residue stress in the indent area, while compressive stress tends to form around the indent to balance the tensile stress generated on the indent. A larger inhomogeneous stress was formed around the edges of the indent imprint in sample-5 (see Figure S4d), similar to the condition of 300 nm depth indent sample (sample-3, see Figure 2i in the main text) which has the same thickness; this is mainly due to asymmetric or un-sharp crack

prolongations formed with the indent fracture thus resulting in an asymmetric stress accumulation.

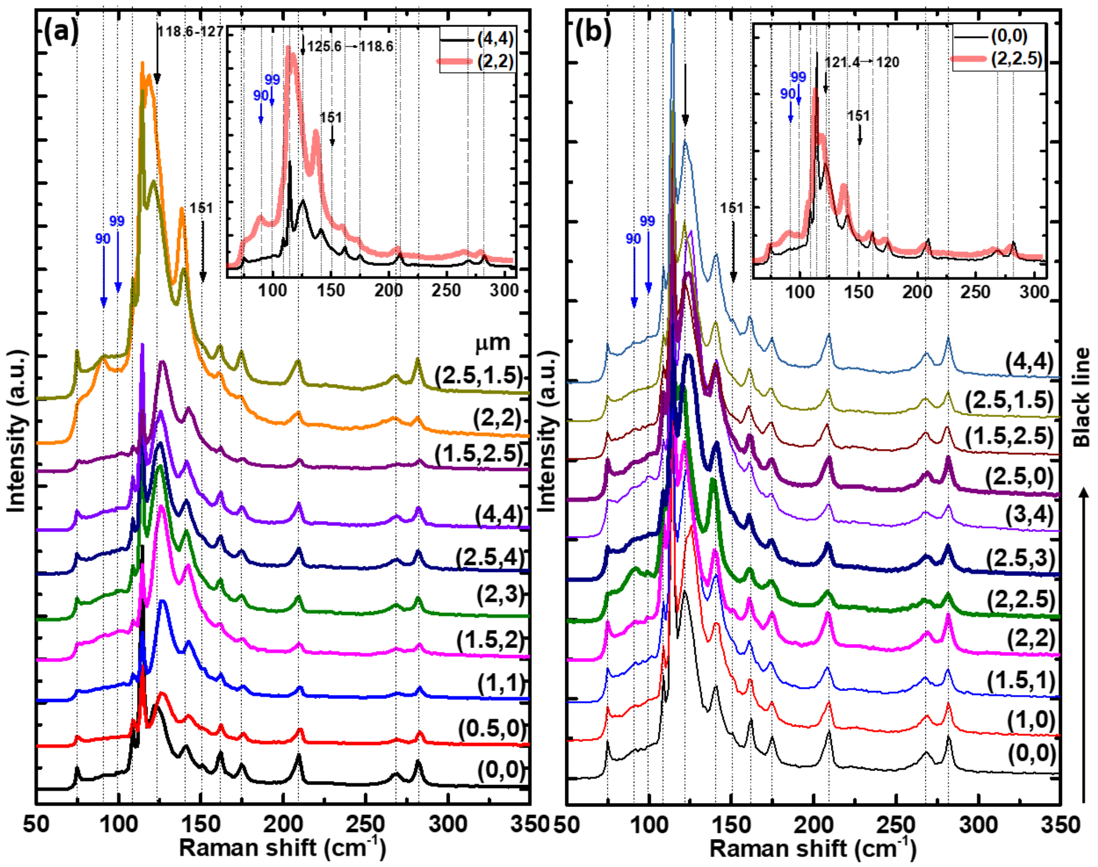

Figure S5. Micro-Raman spectrum evolution along the black line and selected points (as marked in Figure 2 in the main text) after: (a) 80 nm depth indentation (sample-1, corresponding to Figure 2g in the main text), and (b) 250 nm (sample-2, corresponding to Figure 2h in the main text); inset is the detailed Raman spectrum comparison between the indent-center and non-indent area. No significant amorphous-like broadened peaks appeared in the Raman spectrum, while the new peaks around 90 and 99cm^{-1} observed in the near-indent-center region (as shown in insets) which are similar to those features discussed in the main text, likely the consequence from a local amorphization like structure transformations.

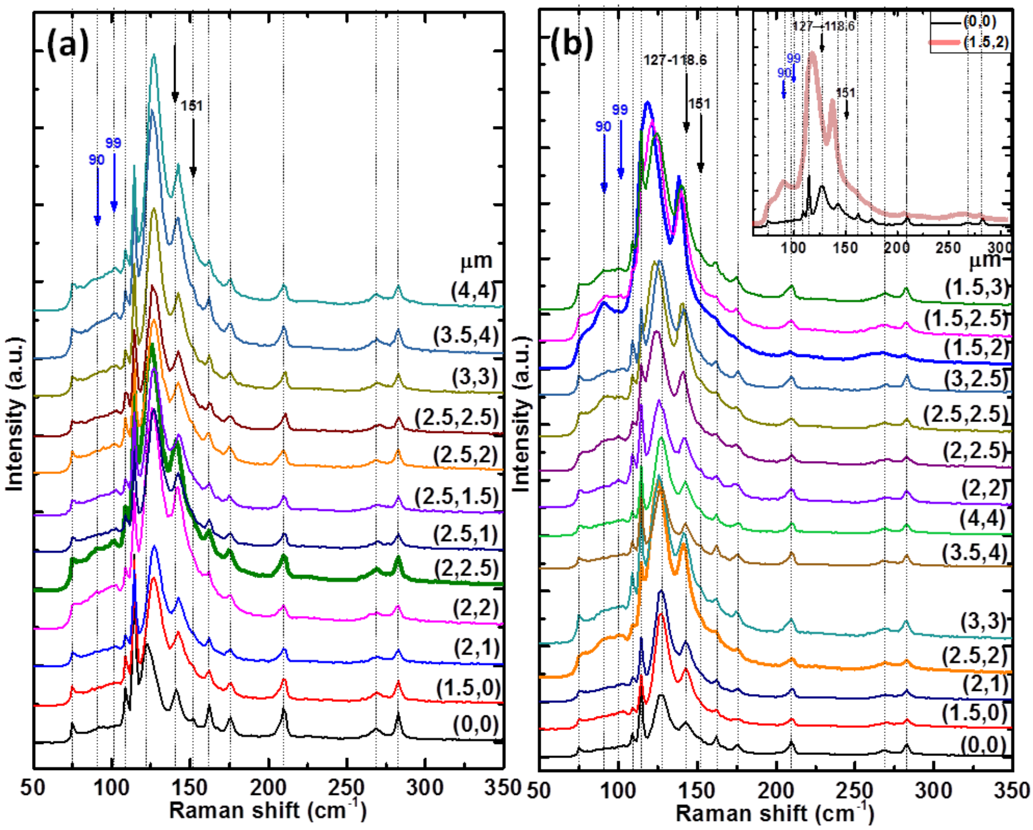

Figure S6. Micro-Raman spectrum evolution along the black line and selected points marked in Figure S4 for (a) sample-4, and (b) sample-5. Inset in (b) is the detailed Raman spectra comparison of the near-center region and the non-indent region; no significant materials changes happened, while an amorphous like Raman spectrum similar to that of Figure 3 and Figure S5b appeared, indicating a local amorphization like structure transformation was similarly induced.

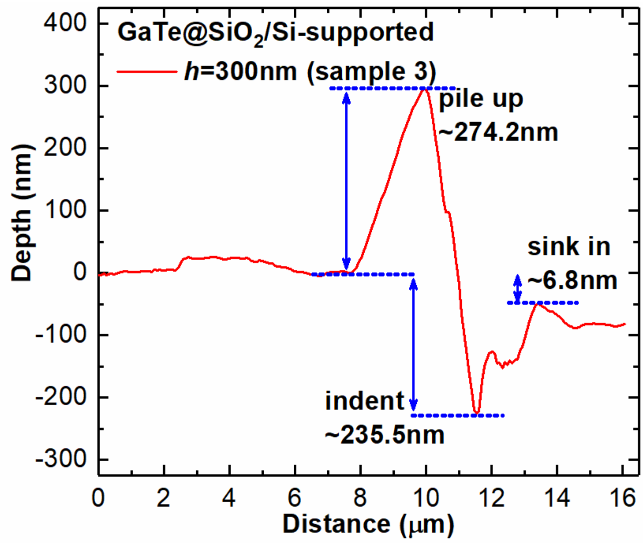

Figure S7. AFM profile measurements on the nanoindentation pit of the 300nm depth (sample-3) indentation sample, with the indent depth, pile up depth and sink in depth labeled, respectively. An indent depth of ~235.5nm was left after the nanoindentation although a 300nm displacement was loaded, and a maximum pile-up of ~274.2nm was resulted while the opposite crack corner presented a slight sink-in depth of ~6.8nm.

5. Supplementary AFM and micro-Raman spectrum of suspended samples

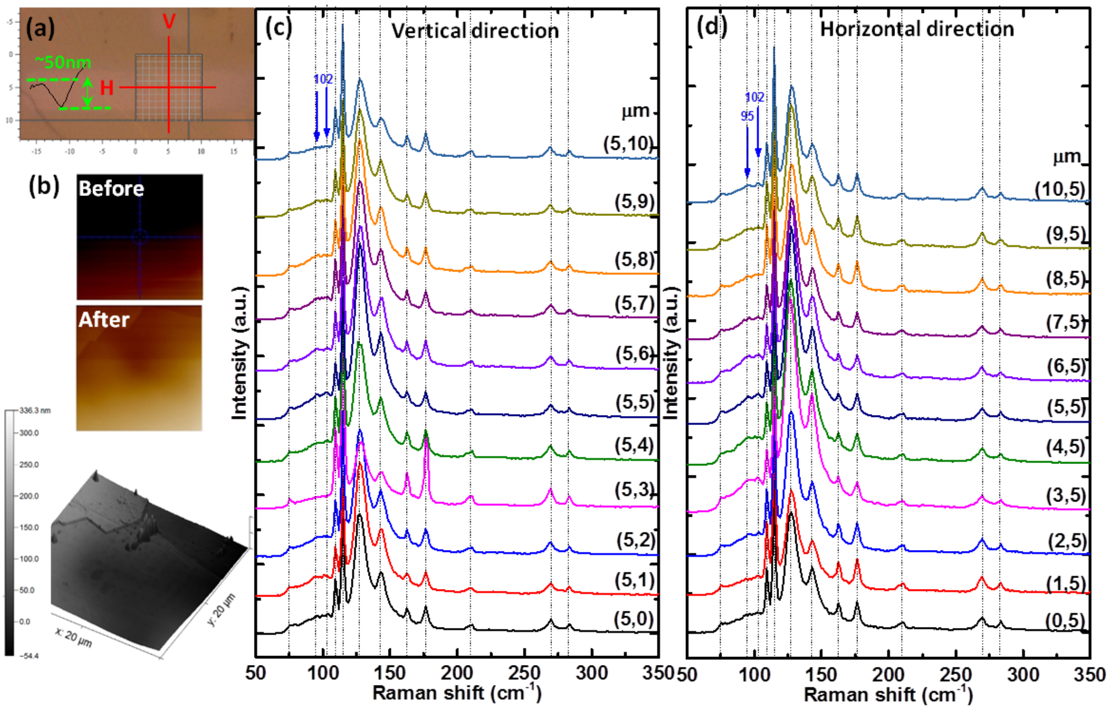

Figure S8. Measurements after 250nm depth (sample-7) indentation on the GaTe multilayers suspended on rectangular membrane slits fabricated on the SiO_2/Si substrate: (a) Optical microscopy image and mapping area; (b) AFM images of the indent topography - top figures indent area phase images before and after the indent, while bottom figure is the 3D topography image of indent area; (c) micro-Raman spectra evolution along the vertical direction (V) and (d) horizontal direction (H) of the mapping area.

From the AFM topography, by plotting the depth profile across the indent region, a permanent concave imprint of ~50nm in depth was left after the nanoindentation, as illustrated by the AFM curve in the inset of Figure S8a and the AFM phase images in Figure S8b, indicating a permanent plastic or unrecoverable deformation. Notably, the asymmetric depth profile may be due to the indentation position not at the center of the rectangular slits (located at ~4.5μm/6μm position of the slits). No observable difference can be seen in the Raman spectra, implying an unchanged sample quality even after nanoindentation of an available maximum depth (250nm in this work).

6. Supplementary Molecular Dynamics simulations

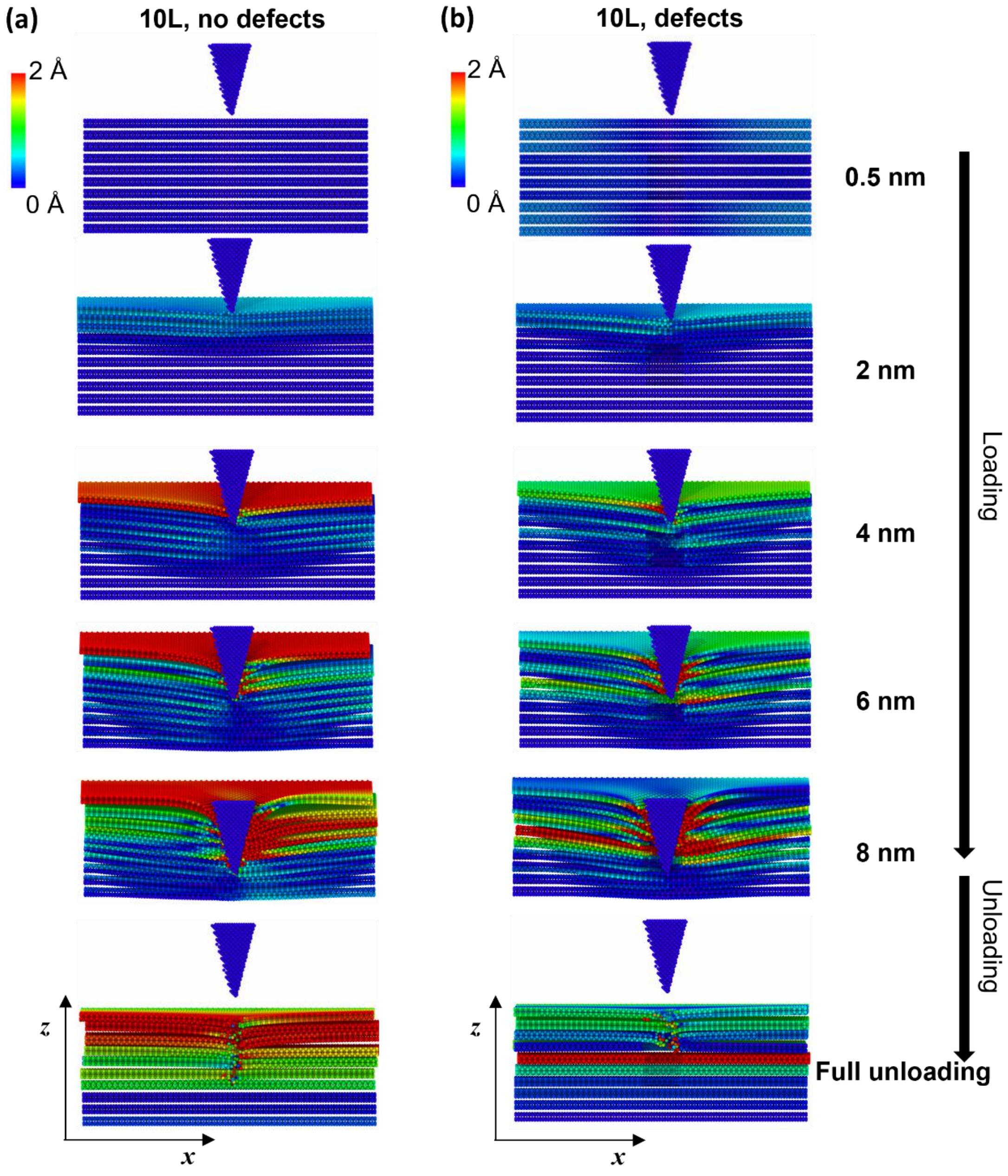

Figure S9. Details of the generated fractures and interlayer sliding along the in-plane *x*-direction at various depth of loading and unloading process for (a) '10L, no defects' samples and (b) '10L, defects' samples obtained in MD simulations.

References:

(1) Castellanos-Gomez, A.; Buscema, M.; Molenaar, R.; Singh, V.; Janssen, L.; Van der Zant, H. S.; Steele, G. A. Deterministic transfer of two-dimensional materials by all-dry viscoelastic stamping. *2D Mater.* **2014,** *1*, 011002.

(2) Mercado, E.; Zhou, Y.; Xie, Y.; Zhao, Q.; Cai, H.; Chen, B.; Jie, W.; Tongay, S.; Wang, T.; Kuball, M. Passivation of layered gallium telluride by double encapsulation with graphene. *ACS Omega* **2019,** *4*, 18002-18010.

(3) Zhang, J.; Zhou, Y.; Ying, P.; Sun, H.; Zhou, J.; Wang, T.; Jie, W.; Kuball, M. Effects of interlayer interactions on the

nanoindentation response of freely suspended multilayer gallium telluride. *Nanotechnology* **2020,** *31*, 165706.

(4) Plimpton, S. Fast parallel algorithms for short-range molecular dynamics. *J. Comput. Phys.* **1995,** *117*, 1-19.

(5) Demirci, S.; Avazlı, N.; Durgun, E.; Cahangirov, S. Structural and electronic properties of monolayer group III monochalcogenides. *Phys. Rev. B* **2017,** *95*, 115409.

(6) Jiang, J.-W.; Zhou, Y.-P. Handbook of Stillinger-Weber potential parameters for two-dimensional atomic crystals. BoD–Books on Demand, IntechOpen, 2017.

(7) Stuart, S. J.; Tutein, A. B.; Harrison, J. A. A reactive potential for hydrocarbons with intermolecular interactions. *J. Chem. Phys.* **2000,** *112*, 6472-6486.

(8) Tersoff, J. Modeling solid-state chemistry: Interatomic potentials for multicomponent systems. *Phys. Rev. B* **1989,** *39*, 5566.

(9) Munetoh, S.; Motooka, T.; Moriguchi, K.; Shintani, A. Interatomic potential for Si–O systems using Tersoff parameterization. *Comput. Mater. Sci.* **2007,** *39*, 334-339.

(10) Rappé, A. K.; Casewit, C. J.; Colwell, K.; Goddard III, W. A.; Skiff, W. M. UFF, a full periodic table force field for molecular mechanics and molecular dynamics simulations. *J. Am. Chem. Soc.* **1992,** *114*, 10024-10035.

(11) Chandrachud, P. Thermodynamics of confined gallium clusters. *J. Phys.: Condens. Matter* **2015,** *27* (44), 445502.

(12) Kinney, K. E.; Bartell, L. S. Potential function for tellurium hexafluoride molecules in the solid. *J. Phys. Chem.* **1996,** *100*, 15416-15420.

(13) Robinson, I.; Tweet, D. Surface X-ray diffraction. *Rep. Prog. Phys.* **1992,** *55*, 599-651.
